# Correlation of histopathologic characteristics to protein expression and function in malignant melanoma

**DOI:** 10.1371/journal.pone.0176167

**Published:** 2017-04-26

**Authors:** Charlotte Welinder, Krzysztof Pawłowski, A. Marcell Szasz, Maria Yakovleva, Yutaka Sugihara, Johan Malm, Göran Jönsson, Christian Ingvar, Lotta Lundgren, Bo Baldetorp, Håkan Olsson, Melinda Rezeli, Thomas Laurell, Elisabet Wieslander, György Marko-Varga

**Affiliations:** 1 Division of Oncology and Pathology, Dept. of Clinical Sciences Lund, Lund University, Lund, Sweden; 2 Centre of Excellence in Biological and Medical Mass Spectrometry “CEBMMS”, Biomedical Centre D13, Lund University, Lund, Sweden; 3 Faculty of Agriculture and Biology, Dept. of Experimental Design and Bioinformatics, Warsaw University of Life Sciences, Warszawa, Poland; 4 Dept. of Translational Medicine, Lund University, Malmö, Sweden; 5 2nd Dept. of Pathology, Semmelweis University, Budapest, Hungary; 6 Dept. of Surgery, Dept. of Clinical Sciences Lund, Lund University, Skåne University Hospital, Lund, Sweden; 7 Dept. of Oncology, Skåne University Hospital, Lund, Sweden; 8 Cancer Epidemiology, Dept. of Clinical Sciences, Lund University, Lund, Sweden; 9 Clinical Protein Science & Imaging, Biomedical Centre, Dept. of Biomedical Engineering, Lund University, BMC D13, Lund, Sweden; 10 First Dept. of Surgery, Tokyo Medical University, Tokyo, Japan; Medical University of Gdańsk, POLAND

## Abstract

**Background:**

Metastatic melanoma is still one of the most prevalent skin cancers, which upon progression has neither a prognostic marker nor a specific and lasting treatment. Proteomic analysis is a versatile approach with high throughput data and results that can be used for characterizing tissue samples. However, such analysis is hampered by the complexity of the disease, heterogeneity of patients, tumors, and samples themselves. With the long term aim of quest for better diagnostics biomarkers, as well as predictive and prognostic markers, we focused on relating high resolution proteomics data to careful histopathological evaluation of the tumor samples and patient survival information.

**Patients and methods:**

Regional lymph node metastases obtained from ten patients with metastatic melanoma (stage III) were analyzed by histopathology and proteomics using mass spectrometry. Out of the ten patients, six had clinical follow-up data. The protein deep mining mass spectrometry data was related to the histopathology tumor tissue sections adjacent to the area used for deep-mining. Clinical follow-up data provided information on disease progression which could be linked to protein expression aiming to identify tissue-based specific protein markers for metastatic melanoma and prognostic factors for prediction of progression of stage III disease.

**Results:**

In this feasibility study, several proteins were identified that positively correlated to tumor tissue content including IF6, ARF4, MUC18, UBC12, CSPG4, PCNA, PMEL and MAGD2. The study also identified MYC, HNF4A and TGFB1 as top upstream regulators correlating to tumor tissue content. Other proteins were inversely correlated to tumor tissue content, the most significant being; TENX, EHD2, ZA2G, AOC3, FETUA and THRB. A number of proteins were significantly related to clinical outcome, among these, HEXB, PKM and GPNMB stood out, as hallmarks of processes involved in progression from stage III to stage IV disease and poor survival.

**Conclusion:**

In this feasibility study, promising results show the feasibility of relating proteomics to histopathology and clinical outcome, and insight thus can be gained into the molecular processes driving the disease. The combined analysis of histological features including the sample cellular composition with protein expression of each metastasis enabled the identification of novel, differentially expressed proteins. Further studies are necessary to determine whether these putative biomarkers can be utilized in diagnostics and prognostic prediction of metastatic melanoma.

## Introduction

Cutaneous metastatic melanoma (MM) is the sixth most common cancer worldwide with an increasing incidence especially in the Northern European countries and Australia [1, 2]. In Sweden it is the fifth most prevalent malignant neoplasm both in men (following prostate, colon, non-melanocytic skin and urinary tract tumors) and women (following breast, colon, non-melanocytic skin and uterine neoplasms) with an annual incidence of 5% [3, 4].

Malignant melanoma possessing a wide variety of cytomorphologic features can be considered as a challenge from a diagnostic perspective, and is well-known for its metastatic spread, most common to the lungs, liver, brain, bone and skin but sometimes to any, and often unusual location in the body. Melanoma tumors commonly possess a broad toolkit to escape from the body surveillance system [5] thus facilitating further metastatic spread. Examples of such mechanisms are replicative immortality, genome instability and mutation, resistance to cell death, deregulated cellular energetics, sustaining proliferative signaling, evasion of growth suppressors, activation of invasion and metastasis, tumor promoting inflammation and means to avoid immune destruction [6].

Screening and early detection improve survival from MM and a better outcome can be expected once surgical removal of early primary lesion was performed, still about 15–25% of the sentinel lymph nodes contain metastatic melanoma cells [7]. Progression from the lymph nodes may occur with time, and metastatic melanoma has been inherently difficult to treat with a low survival rate (< 15% at 5 years) [8]. A phase 3 clinical trial (Multicenter Selective Lymphadenectomy Trial II, or MSLT-II) is designed and conducted to answer the question whether sentinel lymph node biopsy should routinely be followed by removal of the remaining regional lymph nodes (complete lymph node dissection) if the sentinel lymph node is positive for melanoma, or it should be followed-up by ultrasound [9]

Factors determining prognosis routinely rely on clinicopathological characteristics of the patient and primary tumor [10–13], which have been supplemented with DNA sequencing studies in the past years [14–20]. Most melanomas harbor alterations in the BRAF, NF1, RAS, MDM2 (KIT) genes, which result in activation of the MAPK and RAS pathways conferring survival advantage by reprogramming crucial cell cycle and apoptotic cellular functions [21]. Clinical and pathological properties partly reflect the identified (BRAF, RAS, NF1, triple wild type) genomic subtypes of melanoma, but from both clinical and genetic perspective the groups still are heterogeneous [21].

Newly developed drugs allowing targeted therapy such as kinase inhibitors or drugs modulating the immune response currently provide more promise to the patients [22–27]. However, some of these newer treatments have also been subjected to the development of resistance [28]. Recently, the Food and Drug Administration (FDA) approved nivolumab (Opdivo), a PD-1 immune checkpoint inhibitor, for the treatment of advanced (unresectable or metastatic) melanoma in adults, regardless of BRAF status. The drug was approved by FDA for the treatment of patients both with BRAF V600 wild-type unresectable and BRAF V600 mutation-positive metastatic melanoma in addition to combination treatment with ipilimumab (anti-CTLA-4 antibody). With several options requiring individualized treatment, there is a great demand for validated biomarkers that can support both the primary diagnosis and preferably predict the progression of disease and response to treatment of metastatic disease. Predictive biomarkers of response to immune checkpoint inhibitors are currently under evaluation including but not limited to the lymphocytic infiltration of the tumor (= the immunoscore) [29], PD-L1 expression and or CTLA-4 expression [30].

Building upon our previous proteomics work [31] and related genomics studies [32–34], proteomics data for samples from 10 patients with lymph node metastatic melanoma (PRIDE dataset identifier: PXD001725) were referred to histopathological and clinical work-up and evaluation.

Thus, in this feasibility study, in addition to the deep mining proteomic analysis utilizing high-resolution reversed phase nano-separation in combination with mass spectrometry, we also performed in depth pathological characterization (including tissue composition, tumor characteristics and lymphocytic infiltration). The pathological analyses were performed on tumor tissues sections adjacent to the areas used for deep mining. Clinical follow-up information of the patients was reviewed and the cases were grouped into prognostic sets. Hence, the protein expression could be related to the specific cellular composition of the sample. Based on identified protein signatures, molecular classification and clinicopathological characterization, emerging biological relevance could be assigned to several marker proteins.

## Materials and methods

### Clinical samples

**Fig 1:**
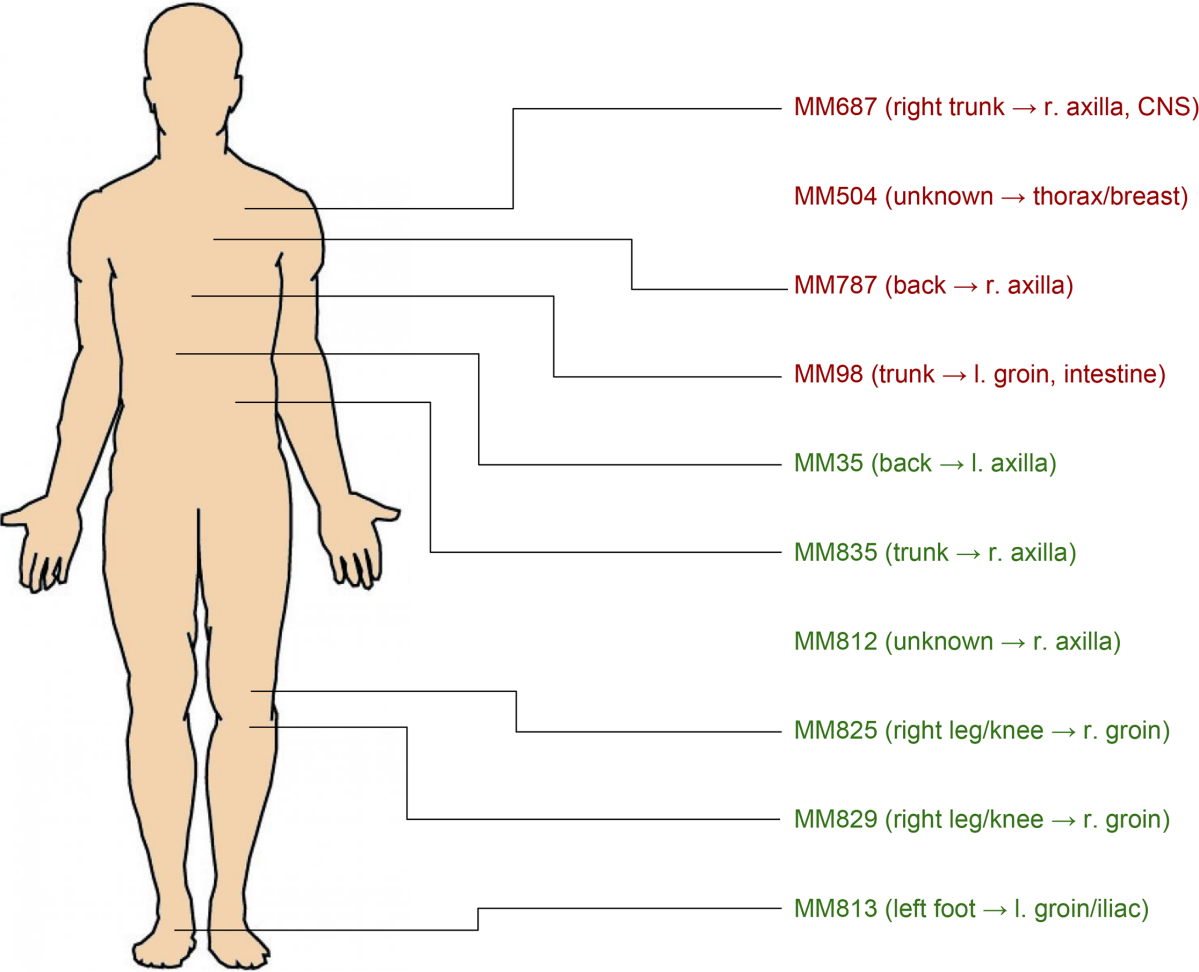
Location of primary tumors on the body of melanoma patients and their route of progression. Red font represents poor prognosis (“non-survivors”), green font represents good prognosis (“survivors”).

The location of primary tumors and progression of melanomas is displayed in Fig 1. This study was approved by the Regional Ethical Committee at Lund University, Southern Sweden, approval number: DNR 191/2007 and 101/2013. All patients within the study provided a written informed consent. The tumor tissues used were lymph node metastases from 10 melanoma patients undergoing surgery at Lund University Hospital, Sweden. The clinical information on respective patients is summarized in Table 1. The fresh specimens were divided into two parts. One portion of the metastasis was fixed in formalin, embedded in paraffin (FFPE) and subsequently sectioned for histopathologic confirmation and the other part was snap frozen within minutes after removal and stored at -80°C in the Biobank. All FFPE samples were routinely confirmed for diagnosis at the department of surgical pathology. The frozen specimens were used as described below for both protein expression analysis and further histological evaluations.

**Table 1:** Clinical information of patient characteristics. Breslow thickness and Clarks refer to primary melanoma feature.

Tumor	Gender	Age at met	Age at primary	Breslow class	Clark	Stage	Status	OS	DSS
MM35	Male	55	54	3	4	3	Alive	7960	-
MM98	Male	75	73	4	4	3	Dead	519	519
MM504	Male	54	NA	NA	NA	NA	Dead	451	451
MM687	Male	74	72	1	2	3	Dead	591	591
MM787	Male	81	78	2	4	3	Dead	734	734
MM812	Male	51	NA	NA	NA	NA	Alive	5028	-
MM813	Female	54	54	2	3	3	Alive	4913	-
MM825	Female	66	64	2	4	3	Alive	4508	-
MM829	Male	55	49	1	2	3	Alive	4431	-
MM835	Female	36	32	3	3	3	Alive	4353	-

NA–not available

OS–Calculated from date of sample collection to 20160622.

DSS–Calculated from date of sample collection to date of dead in melanoma disease.

### Histological evaluation of tumors

Frozen tissue samples were sectioned on a cryostat into 6 μm thick sections, placed upon glass slides, dried at 37°C for 30 min and fixed with 100% methanol for 5 min. The sections were stained with hematoxylin and eosin (HE) [35]. Histological examination was performed on sections immediately neighboring on both sides those sections that were solubilized and subjected to LC-MS/MS investigation. The tumor samples were analyzed for their contents regarding ratio of neoplastic area, adjacent lymph node area, necrosis and connective tissue including fat, fibrous tissue or other material. The tumor parameters were grouped and scored as referenced [21]: tumor cell size was initially measured and assigned as < 20 um, 20–25 um, > 25 um; tumor cell shape was classified as epithelioid, spindle or mixed; pigmentation was scored as 0–3 (0 = no melanin pigment, 1 = slight melanin pigmentation visible at high power, 2 = moderate pigmentation visible at low power, 3 = high pigmentation readily visible at low power with dense melanin content); predominant cytoplasmic properties were assigned as unremarkable, eosinophilic or mixed. The lymphocytic infiltration in two dimensions (distribution: 0: no infiltration, 1: less than 25% of tumor infiltrated, 2: 25–50% of tumor infiltrated, 3: more than 50% of tumor infiltrated by lymphocytes; density was assessed on a 0–3 semiquantitative scale) was assessed resulting in a combined (lymphocyte distribution + lymphocyte density) = immunoscore (sum of components: 0, 2–6).

### Prognostic grouping of patients based on clinical follow-up data

**Fig 2:**
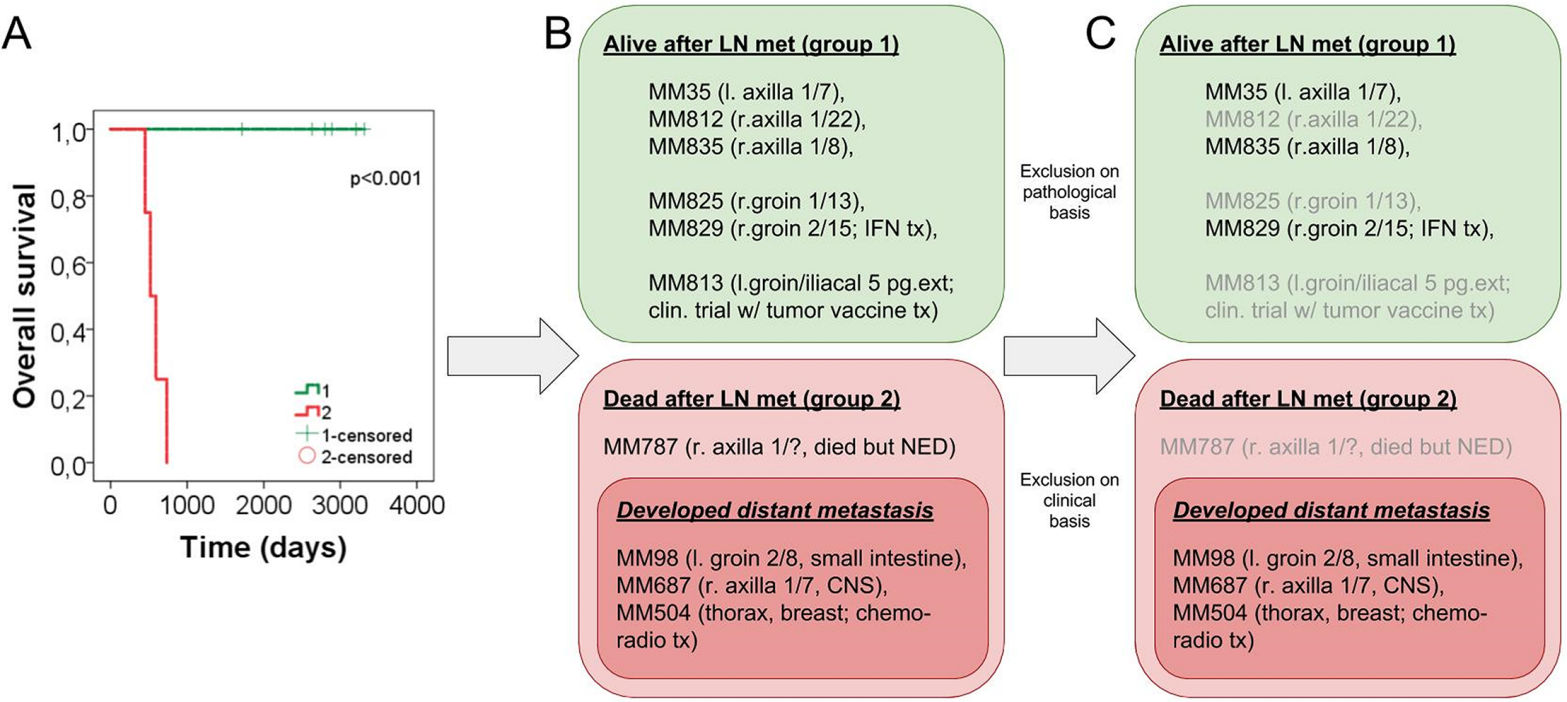
(A) Overall survival of the prognostic groups, “survivors” (group 1) and “non-survivors” (group 2). (B) All ten cases were subjected to rigorous review both on histological and clinical grounds. (C) Those were omitted (lighter grey text), where the tumor content of the examined tissue was low (<10%) or the clinical follow-up data resulted in non-disease-specific outcome measures (one patient in group 2 died without evidence of malignancy). Abbreviations: l: left; r: right; LN met: lymph node metastasis; CNS: central nervous system; tx: therapy. Numbers in brackets mean lymph nodes containing metastatic melanomas / all lymph nodes dissected from either the groin or the axilla: n_metastatic_/n_all_.

For prediction of occurrence of distant organ metastases and eventually, survival we have reviewed the clinical history and the follow-up information on all 10 patients. Overall survival (OS) and disease-specific survival (DSS) of the patients were available. Kaplan-Meier survival curve supported by log-rank test was constructed (Fig 2A). In order to relate protein expression data to clinical outcome, we have identified a group of “survivors” (group 1 in Fig 2A, 2B and 2C), who did not develop any further recurrence of the neoplastic disease on a long-term follow-up period (range = 4353–7960 days) as compared to”non-survivors” (group 2 in Fig 2A, 2B and 2C) who have progressed to stage IV cancer and eventually succumbed to widespread malignant melanoma (range = 451–591 days). We excluded those cases, which had tumor tissue of less than 10% on the slides (Fig 2C), and one case which deceased without evidence of malignancy (Fig 2C). The characteristics of the tumors of the clinically and pathologically stratified patients are displayed in Table 2.

**Table 2:** Histopathological properties of the melanoma metastases evaluated in this study. The ten tumors are grouped according to clinicopathological classification.

GROUPING			Clinicopathologic		
		All	Equivocal*	"survivors"	"non-survivors"
**Tumor cell size**	*<20 microns*	10	4	3	3
** **	*20–25 microns*	0	0	0	0
** **	*>25 microns*	0	0	0	0
**Tumor cell shape**	*epithelioid*	7	2	2	2
** **	*Spindle*	0	0	0	0
** **	*Mixed*	3	1	1	1
**Pigment score**	*0*	2	1	1	0
** **	*1*	2	0	0	1
** **	*2*	2	1	1	0
** **	*3*	4	1	1	2
**Cytoplasm**	*eosinophilic*	10	4	3	3
** **	*Unremarkable*	0	0	0	0
** **	*Mixed*	0	0	0	0
**Lymphocyte distribution**	*0*	0	0	0	0
** **	*1*	4	1	1	2
** **	*2*	3	2	1	1
** **	*3*	1	0	1	0
**Lymphocyte density**	*0*	0	0	0	0
** **	*1*	6	2	1	2
** **	*2*	2	1	1	1
** **	*3*	0	0	0	0
**Immunoscore (= lymphocyte density+ lymphocyte distribution)**	*0*	0	0	0	0
** **	*2*	3	1	1	1
** **	*3*	4	0	1	1
** **	*4*	0	1	0	0
** **	*5*	1	0	1	0
** **	*6*	0	0	0	0
**Tumor (%)**	*Avg*	59.4	21.3	74.6	94.6
**Adjacent lymph node area (%)**	*Avg*	27.0	50.5	21.8	0.5
**Necrosis (%)**	*Avg*	0	0	0	0
**Connective tissue content (%)**	*avg*	13.7	27.8	3.5	4.8

* equivocal cases represent those cases, which had tumor tissue of less than 10% on the slides, and one patient who deceased without evidence of malignancy.

### Proteomics data

Proteomics data were used, as deposited in the PRIDE database, under the dataset identifier PXD001725 [31]. At least two unique peptides were necessary for protein identification, and at the parameter Peptide Confidence in Proteome Discoverer was required to be “high”.

### Bioinformatics

Initial functional analysis of protein lists used the DAVID and ConsensusPathDb tool sets [36, 37]. Enrichment of the lists in particular functional annotations was assessed by Fisher's exact test with Benjamini correction for multiple tests. Gene Ontology annotations, SwissProt keywords, and KEGG pathways were used as annotation terms for the enrichment analysis. For lists of proteins with significantly changed expression, the background protein sets consisted of all proteins detected. Other visualizations, list manipulations and charts were conducted in Spotfire (Tibco Software, Inc., Palo Alto, CA, USA).

Investigation of correlations between protein expression data and histopathology was performed for histological parameters: 1) tumor percentage, 2) connective tissue percentage, 3) adjacent lymph node area and 4) immunoscore. Herein, for protein expression, detection count was used as proxy for abundance. With triplicate proteomics analysis of every patient sample, detection count ranged from zero to three. Spearman rank correlation was used, in home-made scripts within the R environment.

For statistical analysis of significant differences in protein occurrences between the clinical “survivors” and “non-survivors” patient sample sets, t-test was applied as implemented in the Perseus toolkit (http://www.coxdocs.org/doku.php?id=perseus:start). For the purpose of this test, for each protein total PSM count was used as proxy for protein abundance, thus the t-test compared 9 vs. 9 samples for every protein (3 patients per group times 3 replicates).

For in-depth functional analysis of the set of differentially regulated proteins (“survivors” vs. “non-survivors”) and of the set of proteins correlated significantly with sample tumor content, QIAGEN’s Ingenuity Pathway Analysis (Qiagen, Redwood City, CA, USA) was used to generate relationship networks, and perform additional functional analyses.

## Results and discussion

### Histological characterization

The pathological evaluation is based on general directives of pathological assessment of tumor landmarks which is comparable to other studies previously performed [21], in addition to the detailed pathophysiological disease status that each patient´s tissue represents. The first section of tumor tissue was stained with HE, the adjacent sections were subjected to proteomic analysis, and finally the last section was stained with HE and was also characterized by histology. The general tumor cell content of lymph nodes containing metastasis tissues was assessed, and percentages of identified areas were recorded. The melanoma metastases were also characterized for their cytological appearance: tumor cell size and shape, predominant cytoplasmic appearance in line with pigmentation. A pigment score and lymphocyte distribution and density were assessed resulting in a combined immunoscore as described in Materials and Methods (Table 2).

Our previous genomics study using the same patient samples [32], identified “high immune” and the “pigmentation” subtypes of melanoma metastases. Here, we assessed the tumors independently of their intrinsic genomic subtype. All melanoma metastases were composed of generally small sized tumor cells (< 20 microns) with a predominantly eosinophilic cytoplasm (100%, for both properties). The shape of tumor cells was mostly epithelioid in 7 (70%) cases, while presence of admixed spindle morphology was noted in 3 (30%) cases. Melanin pigment was noted as follows; two score 0, two score 1, two score 2 and four score 3 cases were observed. Immunoscore could only be established in 8 cases, because the amount of tumor was too low (≤10%) in two cases for accurate assessment. The lymphocyte distribution was lower than 25% of the tumor in 4 cases, was more widespread (25–50%) in 3 cases and extensive (>50%) in 1 case. The density of lymphocytes was mostly low (score 1) in 6 cases and moderate (score 2) in 2 cases. The combined immunoscore was 2, 3 and 5 in three, four and one case, respectively (Table 2).

### Correlation of protein expression to histological evaluation

In this study, applying conservative criteria, more than 3000 proteins were detected in metastatic melanoma samples [31]. Investigation of correlations between protein expression data and histopathology was performed for histological parameters: 1) tumor percentage, 2) “background” connective tissue percentage, 3) adjacent lymph node area and 4) tumor immunoscore. Herein, for protein expression, detection count was used as proxy for abundance. With triplicate proteomics analysis of every patient sample, the detection count ranged from zero to three. A downside to this approach was the fact that correlation could not be calculated for 159 proteins detected in each sample.

The correlation of protein expression to tumor percentage and to immunoscore were themselves significantly correlated (r = 0.57, p-value < 10^−5^). Among the 288 proteins significantly positively correlated to tumor percentage (shown in S1 Table), there were four proteins clearly labelled as melanoma markers: melanoma cell adhesion molecule (MUC18) [38], melanoma chondroitin sulfate proteoglycan (CSPG4) [39], melanocyte protein (PMEL, melan A), melanoma-associated antigen D2 (MAGD2). Among melanoma markers and proteins linked to this disease, alpha-synuclein and PMEL were significantly correlated to tumor percentage while S100A1 had correlation close to significance at r = 0.61 (p = 0.06. However, S100B had a low correlation to tumor percentage at r = 0.32 (p = 0.37). As many as 71 proteins were negatively correlated to tumor content, however, these may be specific to non-tumor cell types (connective tissues and/or adjacent lymph node) rather than being specific to lack of tumor shown in S2 Table.

An analysis of functional annotations over-represented among proteins correlated to tumor percentage was performed using the ConsensusPathDB and DAVID systems. Both analyses brought similar conclusions. Aminoacyl-tRNA- biosynthesis tRNA-charging was one of top pathways and biological processes significantly related to the protein set. This outcome was due to the fact that nine aminoacyl-tRNA-synthetases were significantly correlated to tumor percentage. These were those for Gln, Thr, Val, Cys, Asp, Ala, His, Arg and Ile, with the r correlation coefficient ranging from 0.67 to 0.81. Aminoacyl tRNA synthetases are believed to be involved in tumorigenesis presumably involving their additional domains which are not directly responsible to their biosynthetic activities [40]. Recently, one aminoacyl tRNA synthetase was used as a cancer drug target [41]. Other pathways and processes manifested by the proteins correlated to tumor percentage, include mitochondrial proteins (more than 80 among significantly correlated) and N-cadherin signaling (including catenins alpha-1, beta-1 and delta-1).

The set of proteins significantly correlated to tumor percentage was also subjected to Ingenuity Pathway Analysis (IPA) system. Among the “Canonical Pathways” significantly enriched in the protein set, tRNA-charging (p = 2,7E-08, 11 proteins out of 82) and Protein Ubiquitination Pathway (p = 4,7E-08, the set included 18 proteins out of 259 pathway proteins) were most significantly related to the protein set. Other canonical pathways included Clathrin-mediated Endocytosis Signaling, Remodeling of Epithelial Adherens Junctions, Acute Phase Response Signaling, Fatty Acid β-oxidation I, EIF2 Signaling. Most of these pathways were implicated in melanoma [42–44]. Ingenuity Pathway Analysis system also yields deduced upstream regulators of a gene set. In the case of proteins correlated to tumor content, the top upstream regulators were MYC, HNF4A and TGFB1, each regulating more than 50 proteins from the query set. For MYC and TGFB1, their roles in melanoma are well-established [45, 46]. Among the general functional annotations over-represented in the query protein set, Cell Death and Survival, Cellular Growth and Proliferation, and Cellular Movement were most significant.

Another application of IPA was “Core Analysis” that considers the overall network of relationships between all human proteins and extracts sub-networks enriched in query proteins. Here, for the set of proteins correlated to tumor content, three top sub-networks, were enriched in molecules involved in Molecular Transport, Protein Trafficking, Cell Signaling (1^st^ subnetwork, S1A Fig), DNA Replication, Recombination, and Repair, Cellular Assembly and Organization, Cell-To-Cell Signaling and Interaction (2^nd^ subnetwork, S1B Fig) and Cancer, Organismal Injury and Abnormalities (3^rd^ subnetwork, S1C Fig).

### Correlation of protein expression to disease outcome

**Fig 3:**
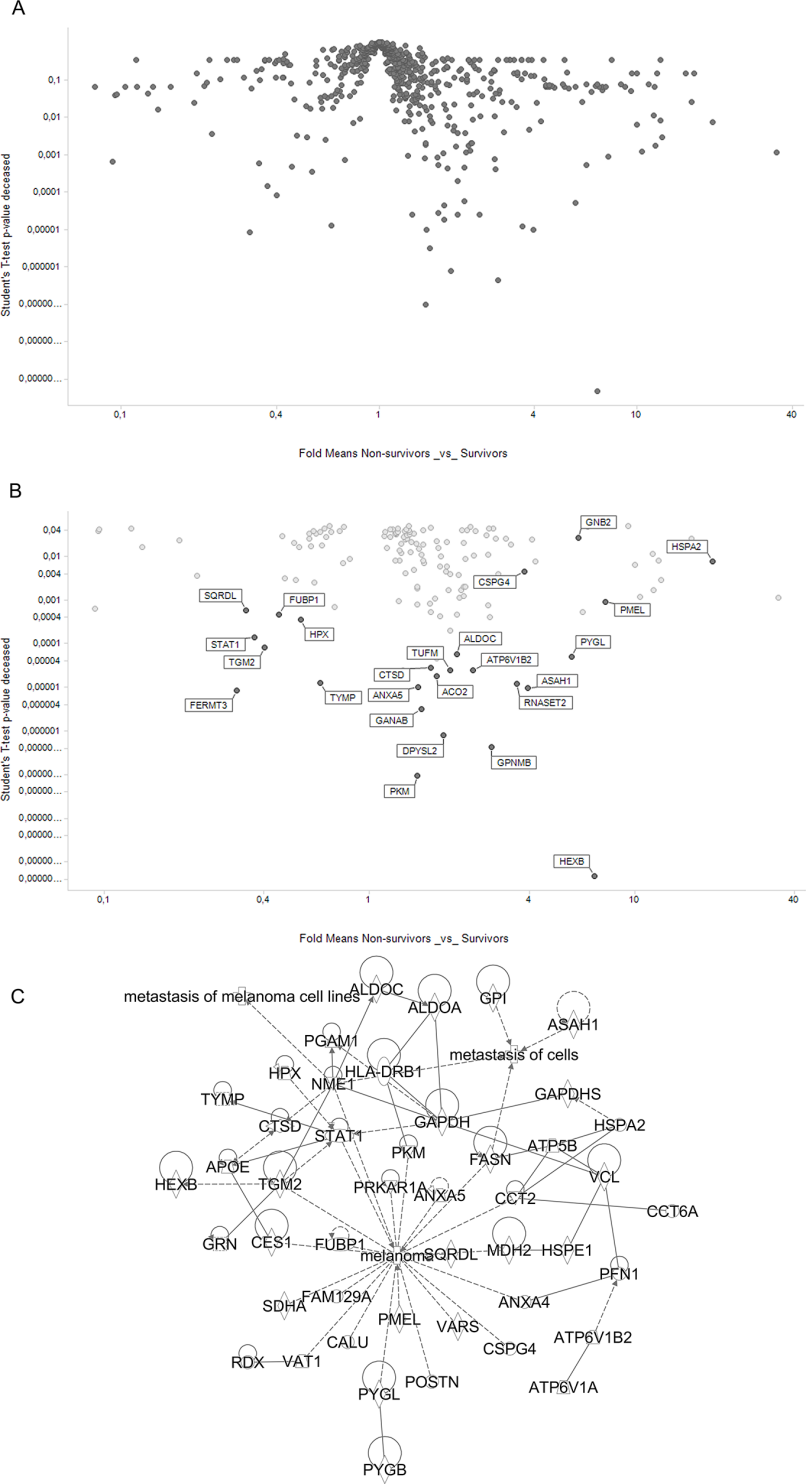
**(A)** Volcano plot showing differences in approximate protein abundance between survivors and non-survivors. T-test p-value and mean fold difference shown (protein abundance evaluated as sum of PSMs). (**B**) As in (A), but only significant proteins shown (p<0.05). (**C)** Biological relationship network (IPA) for proteins differentiating between survivors and non-survivors (shown in B), and having literature links to melanoma or metastasis. Only significant proteins shown (T-test p-value below 0.01), excluding proteins having no IPA relationships within the presented set.

In order to relate, in a currently performed pilot analysis, protein expression in metastatic lymph nodes to patient survival, a t-test was performed, by comparing for every protein, the sum of PSM counts (as proxy for protein abundance) between three survivors and three non-survivors. The results (see Fig 3A and S3 Table) showed a number of significant differences, suggesting that even for these very low patient numbers, some trends can be observed. As many as 36 proteins had the t-test p-value below 0.001, and 74 proteins had p-value below 0.01 (Fig 3B). Majority of significant proteins had higher expression in non-survivors. The only well-established melanoma marker in that group was PMEL (almost 8-fold more abundant in patients who progressed). However, many proteins from this list have previously been linked to melanoma in the literature (Fig 3C).

Strikingly, most significant in relation to patient survival is HEXB, or Beta-hexosaminidase subunit beta. This lysosomal enzyme is involved in hydrolysis of gangliosides GM2 to GM3. A recent study by Tringali et al showed that poor survival in melanoma is significantly related to GM3 levels [47]. These data agree well with our preliminary finding of elevated HEXB levels in patients with poor survival. Another study reported that increased levels of a related ganglioside GD3 increase malignant properties of melanoma cells [48], thus supporting the hypothesis that imbalance in ganglioside forms may contribute to melanoma severity and poor survival. The HEXB protein was also noted by Byrum et al as differing between primary melanoma, benign nevi and MM, but the direction of changes they reported was different [49].

Other proteins most significantly differing between survivors and non-survivors were GPNMB (glycoprotein non-metastatic melanoma protein B, a homologue of PMEL) and PKM (pyruvate kinase, muscle) hemopexin (HPX), complement factors (C3, C4B and CFH) and vinculin (VCL), Annexin A5 (ANXA5), V-type proton ATPase catalytic subunits (ATP6V1A, ATP6V1B2) and two aminoacyl tRNA ligases (VARS and IARS2). GPNMB was shown previously to be linked to metastasis, and is in clinical trials for melanoma [50]. PKM controls glycolysis, a process crucial for metabolism in malignant cells, and the balance of splicing-derived PKM isoforms is known to be related to cancer invasiveness [51, 52]. The Complement C3a Receptor signaling has been very recently shown to contribute to melanoma tumorigenesis [53]. On the contrary, differential expression of vinculin between weakly and highly metastatic melanoma cell lines was noted more than 20 years ago [54].

The IPA analysis was performed for the sets of proteins that differed between survivors and non-survivors (S2 Fig and S3A–S3D Fig). Core IPA analysis revealed that most significant canonical pathway enriched in the set of proteins changed between survivors and non-survivors was glycolysis (p-value 9.8E-13) while cell death and survival, carbohydrate metabolism, cellular movement, and cellular growth and proliferation were the most significant biological functions overrepresented in this protein set. This is consistent with biological processes known to be related to metastatic properties of cancer.

Despite a very small number of patients in this pilot evaluation, (three survivors and three non-survivors), a number of proteins were found to differentiate the two groups, and substantial number of these was previously linked to cancer in the literature (S2 Fig). Among the proteins most significantly differentiating between the two patient groups, HEXB, PKM and GPNMB stood out, as hallmarks of processes involved in melanoma progression and responsible for poor survival.

## Conclusion

**Fig 4:**
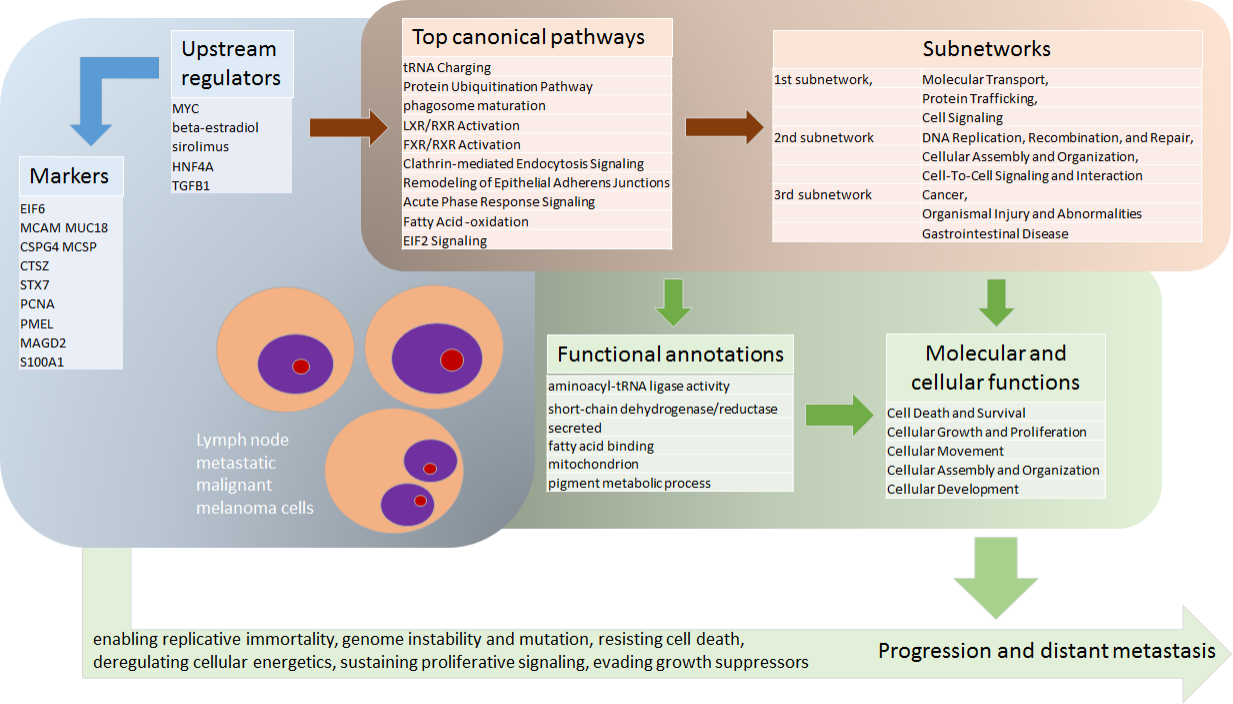
Identified melanoma markers and pathobiological processes in tissue samples of melanoma lymph node metastases. The graphics displays key upstream regulators found according to analysis based on DAVID and IPA evaluation. The graph displays major regulated canonical pathways and subnetworks resulting in cellular functions and molecular fingerprints providing survival advantage for malignant cells with promotion to further progression and metastasis.

An in depth pathological analysis of metastatic melanoma samples used for generating deep mining high-quality mass spectrometry data of protein expression is presented. This feasibility study provides an example how clinical outcome and detailed histopathology analysis related to proteomics discovery data can provide novel insights into the molecular processes driving the disease. The main implication of this study is that melanoma proteomics should be inseparable from histological evaluation performed in the most detailed manner. This unique approach is the main strength of this study while it has obvious limitations arising from relatively limited number of patient samples. The mass spectrometry data represent the initial stage of a protein sequence database for metastatic melanoma. The combined knowledge of the tumor cellular composition with protein expression of each metastasis enables identification of novel, significantly regulated proteins, in melanoma tumor tissue sections. Upon further validation and development these proteins might serve as future diagnostic markers for this cancer. In Fig 4, the key upstream regulators, the major regulated canonical pathways and subnetworks according to analysis based on DAVID and IPA evaluation are summarized. Cellular functions and molecular fingerprints that provide survival advantage for malignant cells with promotion to further disease progression were highly represented in our overall analysis. In addition, several markers previously related to melanoma were seen to correlate to the tissue tumor cell content. Although the translational impact of this feasibility study is not immediate, future studies should confirm the relevance of such proteins as important melanoma landmarks.

## Supporting information

S1 Fig **(A)** First, (B) second and (C) third most significant biological relationship subnetworks resulting from Ingenuity Pathways Analysis (IPA) for the proteins correlated to tumor content. Members of the original list of 359 proteins marked in grey. Magenta outline highlights proteins implicated in cancer according to IPA database.
(TIF)

S2 Fig Subcellular locations and biological relationship network for proteins differentiating between survivors and non-survivors. Only significant proteins were shown (t-test, p-value below 0.01). Known cancer biomarkers marked by magenta outline (IPA). Proteins having no IPA relationships within the presented set are excluded. Red filling: proteins with higher expression in non-survivors.
(TIF)

S3 Fig (A) First, (B) second, (C) third and (D) fourth, respectively, most significant biological relationship subnetworks resulting from Ingenuity Pathways Analysis for the proteins differentiating between survivors and non-survivors. Only significant proteins used in the IPA analysis (T-test p-value below 0.01). Known cancer biomarkers marked by magenta outline. Red filling: proteins with higher expression in non-survivors.
(TIF)

S1 Table Proteins significantly positively correlated to tumor content. (XLSX)

S2 Table Proteins significantly negatively correlated to tumor content. (XLSX)

S3 Table Correlation of protein expression to disease outcome. Student's t-test p-value provided for comparison of survivors vs non-survivors. PSM counts compared, three patients in a group, each in triplicate, hence 9 samples per group.
(XLSX)
