# Supplementary figures and images for: Correlation of histopathologic characteristics to protein expression and function in malignant melanoma
Source: PLoS One. 2017 Apr 26;12(4):e0176167. doi: 10.1371/journal.pone.0176167 (PMC5405986; doi:10.1371/journal.pone.0176167)

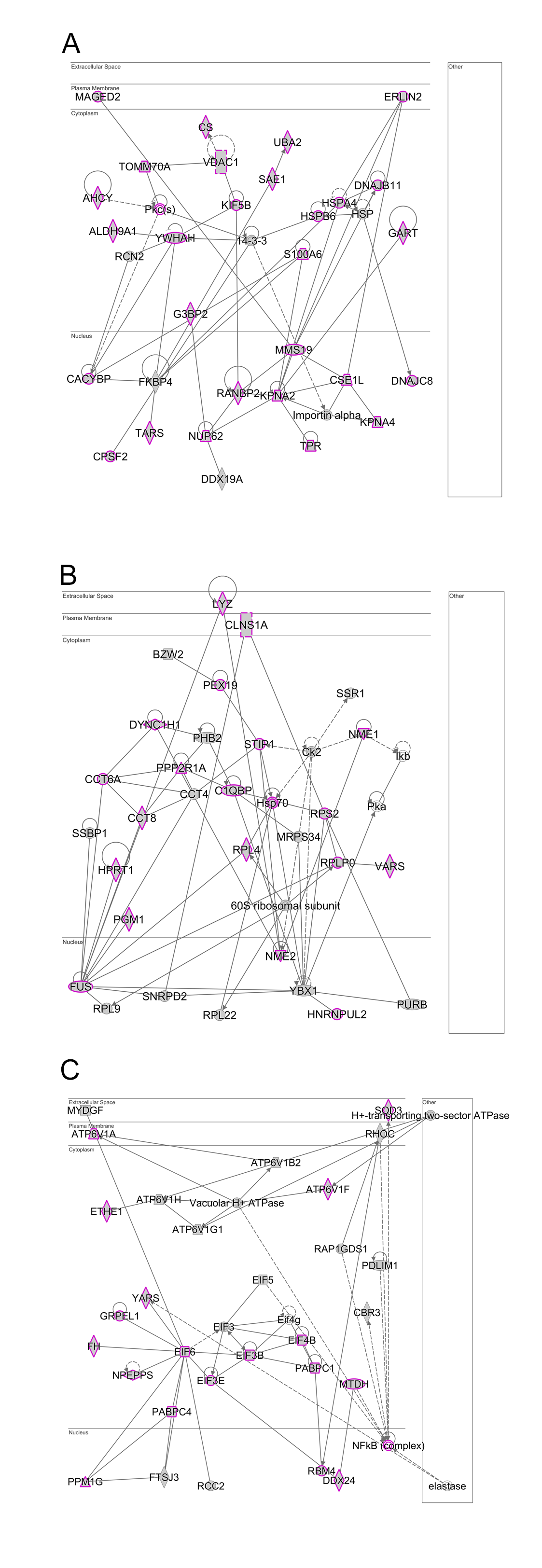

Supplement: S1 Fig — (A) First, (B) second and (C) third most significant biological relationship subnetworks resulting from Ingenuity Pathways Analysis (IPA) for the proteins correlated to tumor content. Members of the original list of 359 proteins marked in grey. Magenta outline highlights proteins implicated in cancer according to IPA database. (TIF) [file pone.0176167.s001.tif]

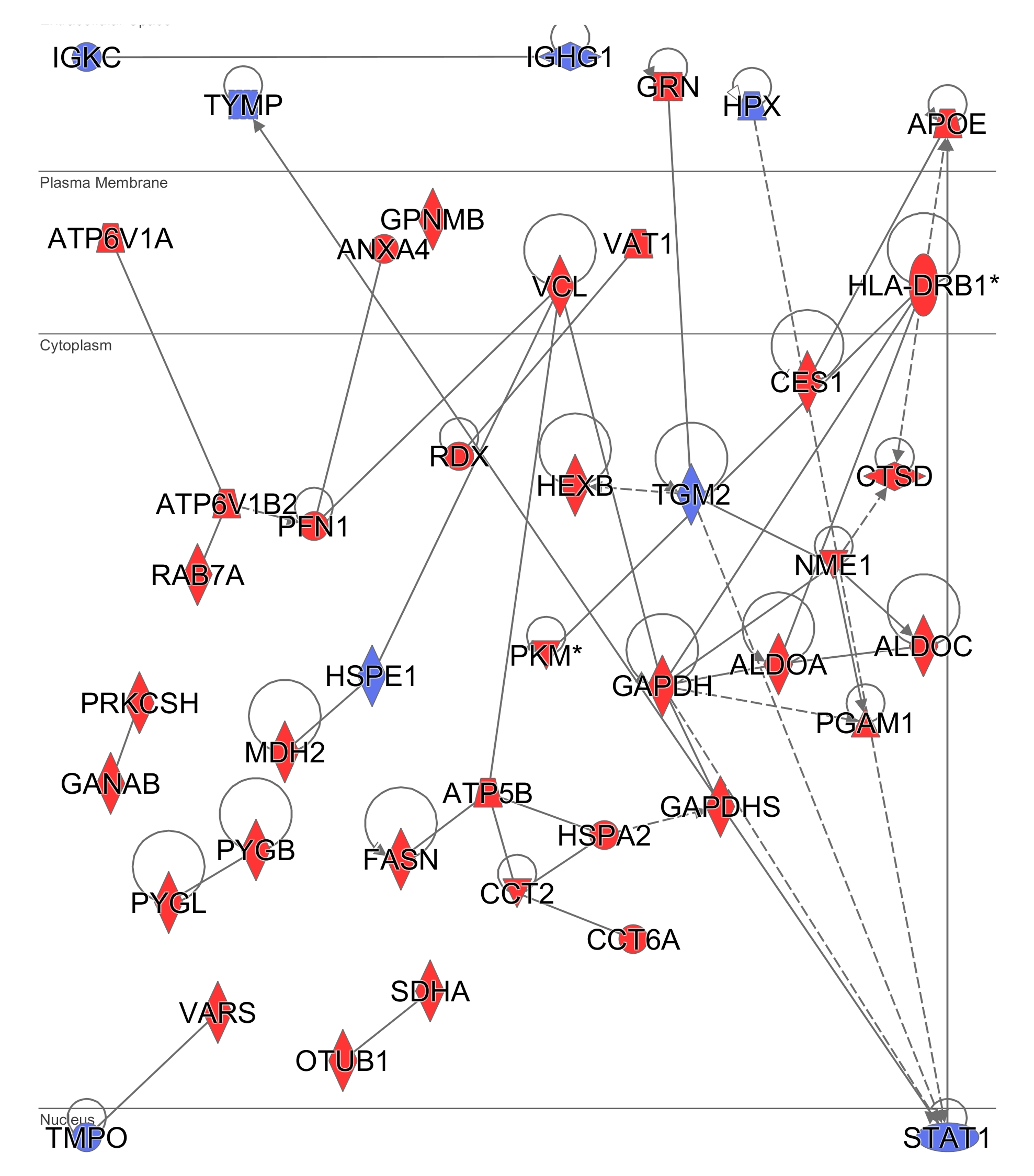

Supplement: S2 Fig — Only significant proteins were shown (t-test, p-value below 0.01). Known cancer biomarkers marked by magenta outline (IPA). Proteins having no IPA relationships within the presented set are excluded. Red filling: proteins with higher expression in non-survivors. (TIF) [file pone.0176167.s002.tif]

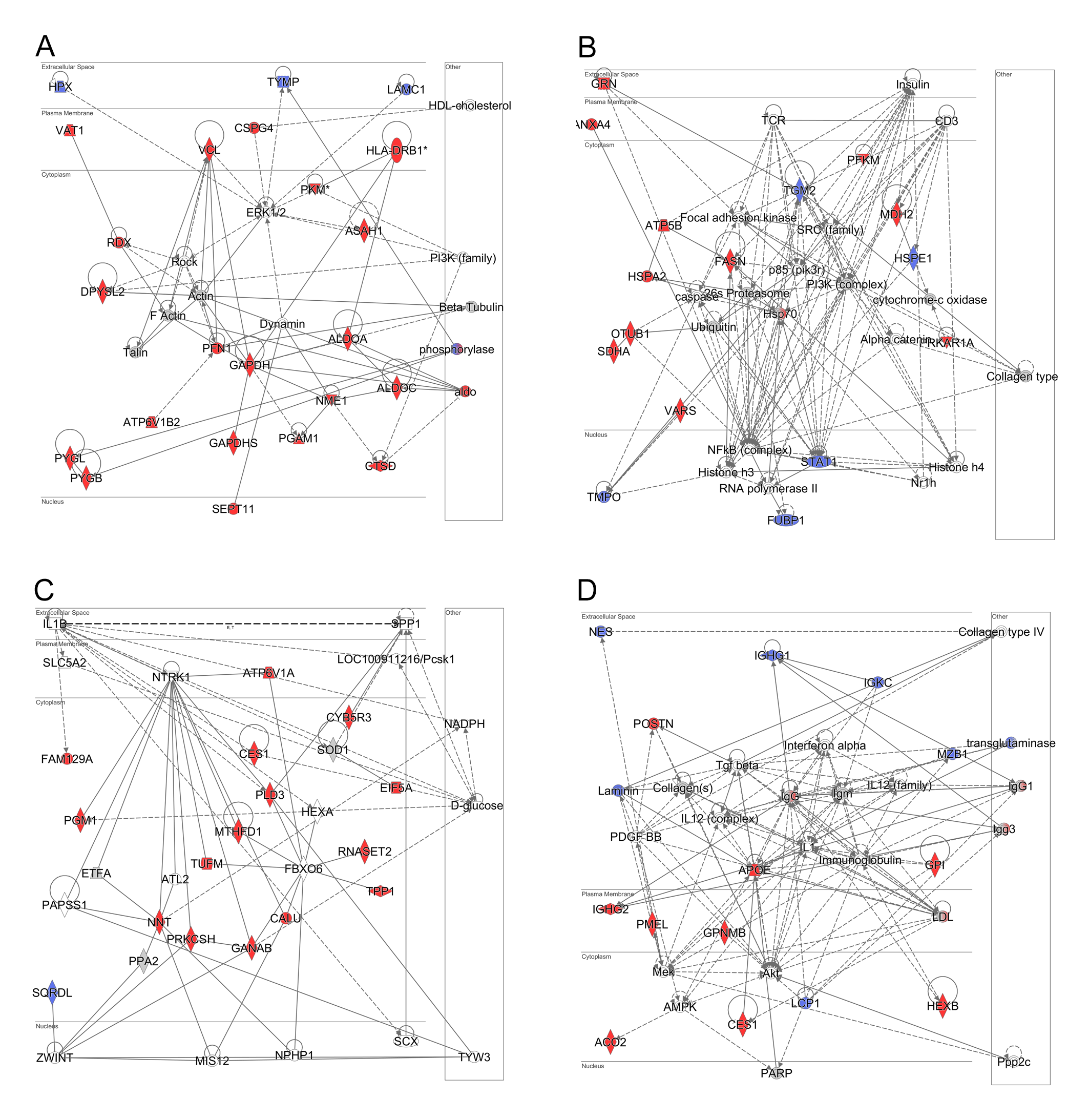

Supplement: S3 Fig — (A) First, (B) second, (C) third and (D) fourth, respectively, most significant biological relationship subnetworks resulting from Ingenuity Pathways Analysis for the proteins differentiating between survivors and non-survivors. Only significant proteins used in the IPA analysis (T-test p-value below 0.01). Known cancer biomarkers marked by magenta outline. Red filling: proteins with higher expression in non-survivors. (TIF) [file pone.0176167.s003.tif]
